# Binder-free sheet-type all-solid-state batteries with enhanced rate capabilities and high energy densities

**DOI:** 10.1038/s41598-018-19398-8

**Published:** 2018-01-19

**Authors:** Mari Yamamoto, Yoshihiro Terauchi, Atsushi Sakuda, Masanari Takahashi

**Affiliations:** 1 Osaka Research Institute of Industrial Science and Technology, Morinomiya Center, 1-6-50, Morinomiya, Joto-ku, Osaka-city, Osaka 536-8553 Japan; 2 Department of Applied Chemistry, Osaka Prefecture University, 1-1 Gakuen-cho, Naka-ku, Sakai, Osaka 599-8531 Japan; 3 Graduate School of Materials Science, Nara Institute of Science and Technology, 8916-5 Takayama-cho, Ikoma, Nara 630-0192 Japan

## Abstract

All-solid-state batteries using inorganic solid electrolytes are considered promising energy storage systems because of their safety and long life. Stackable and compact sheet-type all-solid-state batteries are urgently needed for industrial applications such as smart grids and electric vehicles. A binder is usually indispensable to the construction of sheet-type batteries; however, it can decrease the power and cycle performance of the battery. Here we report the first fabrication of a binder-free sheet-type battery. The key to this development is the use of volatile poly(propylene carbonate)-based binders; used to fabricate electrodes, solid electrolyte sheets, and a stacked three-layered sheet, these binders can also be removed by heat treatment. Binder removal leads to enhanced rate capability, excellent cycle stability, and a 2.6-fold increase in the cell-based-energy-density over previously reported sheet-type batteries. This achievement is the first step towards realizing sheet-type batteries with high energy and power density.

## Introduction

Storage batteries are key to enabling the use of renewable energy and smart grids, but satisfying the requirements for capability, energy and power density, compactness, durability, and safety for next-generation batteries is a major challenge. Conventional lithium-ion batteries (LIBs) that utilize organic liquid electrolytes have been commercially successful in compact portable electronics^1^. However, LIBs can be hazardous because of their flammability and the potential for electrolyte leakage, which is problematic as battery size increases^2^. In contrast, all-solid-state lithium-ion batteries (ASSLBs) using non-flammable inorganic sulfide solid electrolytes (SEs)^3–6^ are promising for large-scale energy storage applications because of their safety, cycling longevity^7–9^, and high power density^10^.

The majority of sulfide-based ASSLBs have been fabricated as pellet-type batteries by powder compression^7–11^. Unfortunately, these batteries have previously only achieved outstanding performance with reduced active material fractions in the composite electrode (40–70 wt.% LiCoO_2_)^7–11^, which maximizes the active material’s performance. Further, powder compression is not optimal for reducing the thickness of the SE layer separator (240–500 μm)^7–11^. Thus, these batteries have been limited by low cell-based-energy -densities (10–45 Wh kg^−1^)^7–11^ compared to those of conventional LIBs (100-200 Wh kg^−1^). Stackable and compact sheet-type batteries are now urgently sought for use in electric vehicles to reduce the cell packaging and wiring used in conventional LIBs^12–17^. A coating process has advantages for the fabrication of sheet-type batteries; coating is scalable and uses conventional fabrication equipment, allowing increased battery size. Binders are indispensable for the dispersion of active materials in slurries and to allow sheet-like morphologies to be produced^12,14–17^. However, binders decrease cell performance by increasing internal cell resistance^14,15^, which impairs the inherent features and advantages of sulfide-based ASSLBs^12^.

We developed sheet-type batteries using a volatile poly(propylene carbonate)-based binder, which is removable after fabrication, and anisole, which satisfies the conflicting demands of solvating the binder and maintaining the SE properties. Adjusting the electrode binder content and removing the binder minimizes the internal cell resistance, which reaches values like those of binder-free pellet-type batteries. Thus, binder removal effectively improves rate capability and long-term stability. We also describe efforts to improve the cell-based-energy-density of binder-free sheet-type full-cells using thinner SE sheets and thicker electrode sheets with high active material loadings.

## Results

### Binder-removal strategy

**Figure 1:**
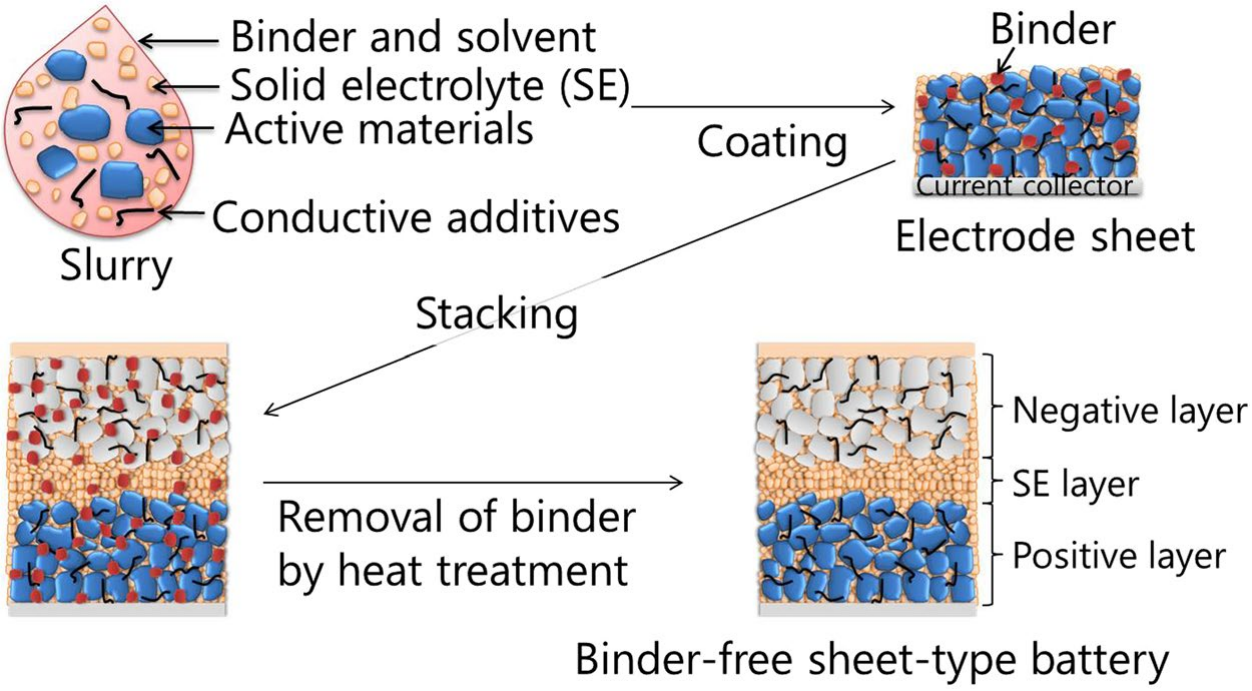
Strategy for the fabrication of binder-free sheet-type ASSLBs. Schematic diagram showing the fabrication process for binder-free sheet-type ASSLBs.

Figure 1 illustrates the fabrication strategy for binder-free sheet-type batteries. Both binder and solvent are used to disperse the SE, active materials, and conductive additives; to maintain a sheet morphology in the positive and negative electrodes and SE sheet, and to prepare three-layered sheets by stacking. Finally, the binder is removed from the three-layered sheet by heat treatment.

Initially, 75Li_2_S·25P_2_S_5_ (mol%)^5^ (LPS) glass was selected as the SE to carry out these processes because of its wide potential window^5^, excellent reduction resistance for Li metal^5^, relatively high ionic conductivity, ~10^−4^ S cm^−1^ ^5^, high chemical stability to moisture^18^ and suitable plastic deformation^19,20^. LPS glass changes to a glass ceramic at 230 °C, and the resulting crystal structure is maintained until 300 °C^5^; thus, we selected aliphatic polycarbonates as volatile binders, which thermally depolymerize by unzipping and evaporate at temperatures below 300 °C (see Supplementary Fig. S1)^21^. Therefore, carbon is removed even when heated under an oxygen-free atmosphere.

### Exploration of solvent and aliphatic polycarbonates

**Figure 2:**
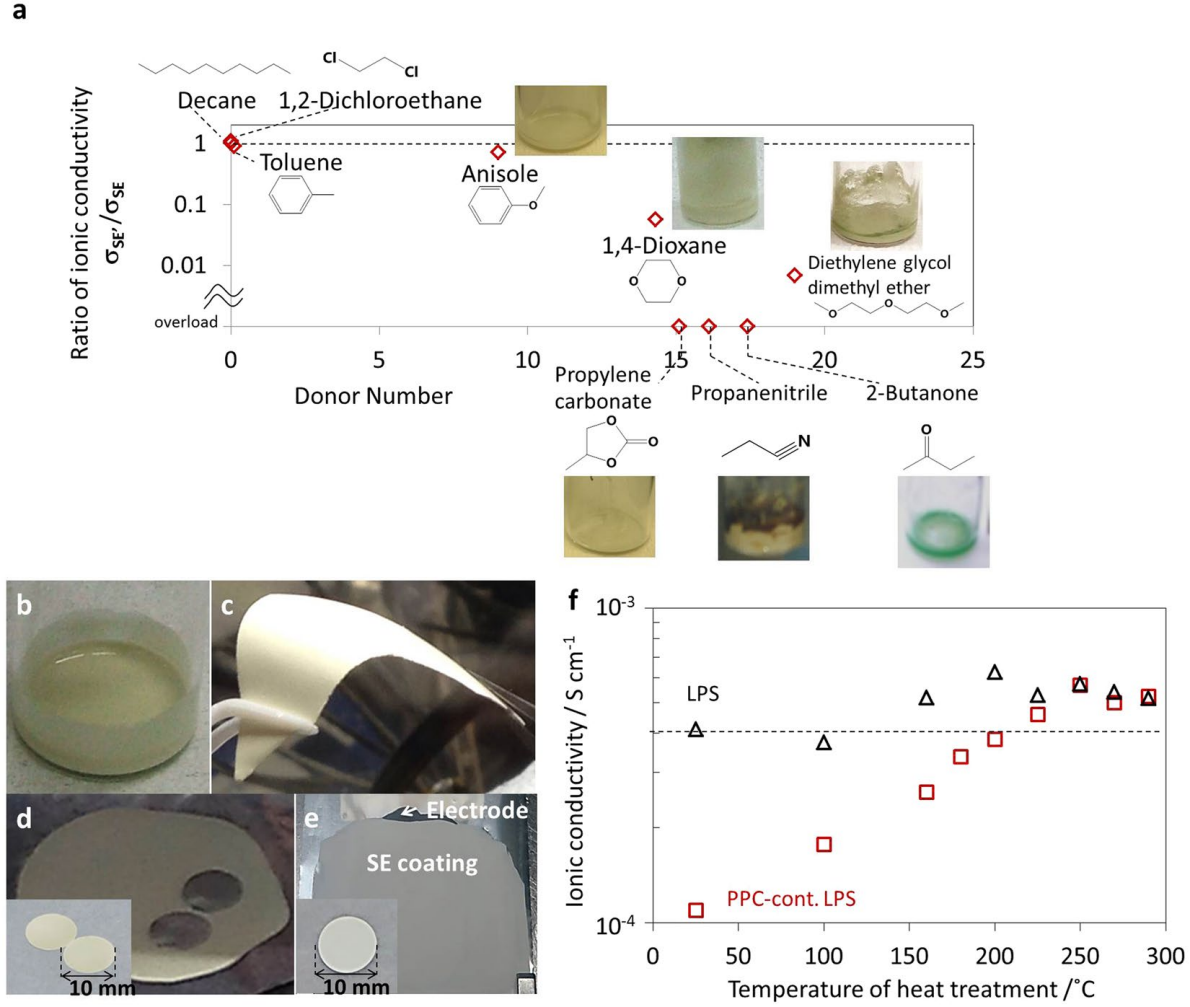
Exploration of solvent and aliphatic polycarbonate properties for the fabrication of binder-free sheet-type batteries. (**a**) Ionic conductivity of solvent-exposed LPS (**σ**_**SE’**_) divided by that of pristine LPS (**σ**_**SE**_), i.e. **σ**_**SE’**_/**σ**_**SE**_, as a function of the solvent’s donor number, where a higher donor number indicates higher nucleophilicity. Images of (**b**) a PPC-containing SE slurry, (**c**) a flexible and free-standing PPC-containing SE sheet (PPC; 6 wt.%, 8 vol%), (**d**) PPC-containing SE sheets punched by pressing a stainless-steel rod under 260 MPa and (**e**) a two-layered sheet prepared by coating the SE slurry on the electrode sheet. The inset is a two-layered sheet cut by punching machine. (**f**) Ionic conductivities of LPS and 6 wt.% PPC-containing LPS powder treated at various temperatures in the range from 25 to 290 °C.

The requirements for a good solvent are that it should not affect the ionic conductivity of LPS, should dissolve the binder, and have a moderate vapor pressure. The ionic conductivities of LPS after exposure to various solvents were investigated to evaluate the stability of LPS (Fig. 2a). As the solvent donor number^22^ is increased to values greater than 14, solvent-exposed LPS shows a significantly decreased ionic conductivity and its color changes from pale yellow to brown and green. LPS became sticky after exposure to propylene carbonate (PC), with an ionic conductivity that was too low to measure. In contrast, LPS showed negligible changes in color and ionic conductivity when exposed to solvents with a donor number of 9 or less, such as anisole, toluene, 1,2-dichloroethane, and *n*-decane. Since solvents with higher donor numbers have higher electron-donating abilities, we conclude that solvents with donor numbers greater than 14 decomposed LPS by nucleophilic attack, causing a decrease in ionic conductivity.

We investigated the solubility of aliphatic polycarbonates in solvents with donor numbers of 9 or less (see Supplementary Table S1 online). Poly(ethylene carbonate) (PEC) dissolved only in 1,2-dichloroethane. Positive sheets prepared with PEC/1,2-dichloroethane had a whitish surface, implying that lightly pulverized fine 75Li_2_S·25P_2_S_5_ glass powder (f-LPS) floated while sedimentation occurred for heavy LiNbO_3_-coated LiNi_1/3_Co_1/3_Mn_1/3_O_2_ (NCM). Moreover, many bulges were observed on the surface of the positive sheets due to the rapid evaporation of 1,2-dichloroethane, which has a high vapor pressure (8.7 kPa, 20 °C)^23^ (see Supplementary Table S2 online). In contrast, poly(propylene carbonate) (PPC) dissolved in both 1,2-dichloroethane and anisole. Positive sheets prepared using PPC/anisole had smooth surfaces with uniform color, implying the homogeneous dispersion of NCM and f-LPS. The slow evaporation of anisole, the vapor pressure of which is low (0.47 kPa, 25 °C)^23^, prevents the segregation of NCM and f-LPS and the formation of cracks and bulges during the drying process. It is worth noting that positive sheets with a pre-compression thickness of 200–300 μm (60–75 μm after compression) were difficult to break by punching even 5 months after fabrication (see Supplementary Fig. S2). Thus, we selected a combination of PPC and anisole for subsequent experiments.

A PPC-containing SE slurry (Fig. 2b) enables the fabrication of bendable free-standing SE sheets (Fig. 2c), which can be punched to afford SE disks (Fig. 2d), and the fabrication of two-layered SE/electrode sheets by slurry coating (Fig. 2e).

Since PC exposure causes a significant decrease in the ionic conductivity of LPS (Fig. 2a), the PC that arises from the depolymerization of PPC (see Supplementary Fig. S1) must be removed from the as-prepared cells quickly under vacuum to minimize the exposure time. We studied the electrochemical properties and the structure of PPC-containing LPS before and after removal of PPC by vacuum heating. Figure 2f shows the ionic conductivity of both pristine and 6 wt.% PPC-containing LPS as a function of the heat treatment temperature. The ionic conductivity of pristine LPS, 4.1 × 10^−4^ S cm^−1^ increased slightly to 5.2 × 10^−4^ S cm^−1^ after heat treatment at temperatures between 160–290 °C^5^. Compared to pristine LPS glass, the conductivity of PPC-containing LPS (PPC; 6 wt.%) decreased significantly to 1.1 × 10^−4^ S cm^−1^, which suggests that non-conductive PPC interferes with ionic conduction at the interface between LPS particles. The ionic conductivity of PPC-containing LPS gradually increased with heat-treatment temperature, returning to that of pristine LPS between 225–290 °C. Even though the sheet size increased from 10 mm to 40 mm in diameter and PPC content increased from 6 wt.% to 10 wt.%, their conductivities returned to that of pristine LPS after heat treatment at 225 °C. Thus, we concluded that heat treatment at 225 °C is sufficient to decompose PPC and remove PC from PPC-containing LPS. Since both pristine and PPC-containing LPS show a similar color change from pale yellow to dark brown with increasing temperatures (see Supplementary Fig. S3), this change was attributed to the heat treatment rather than the removal of PPC.

We confirmed that PPC-containing LPS before and after heat treatment maintains both a framework structure containing PS_4_^3−^ moieties^24^ (see Supplementary Fig. S4a) and a wide electrochemical window of 5 V (see Supplementary Fig. S4b). DC conductivities were evaluated for both pristine and PPC-containing LPS before and after heat treatment to investigate whether the electronic conductivity of LPS is affected by the carbon arising from the decomposition of PPC under an oxygen-free atmosphere (see Supplementary Fig. S4c). While the total conductivity (including ionic and electronic contributions) of all samples were close to those obtained by AC impedance measurements, the electronic conductivities were ~4 orders of magnitude lower than the corresponding total conductivities, suggesting that residual carbon in the LPS layer had a negligible effect. These results demonstrate that the original ionic and electronic conductivity of LPS are retained after the PPC removal under vacuum heat treatment.

### Effect of binder removal on cell performance

**Figure 3:**
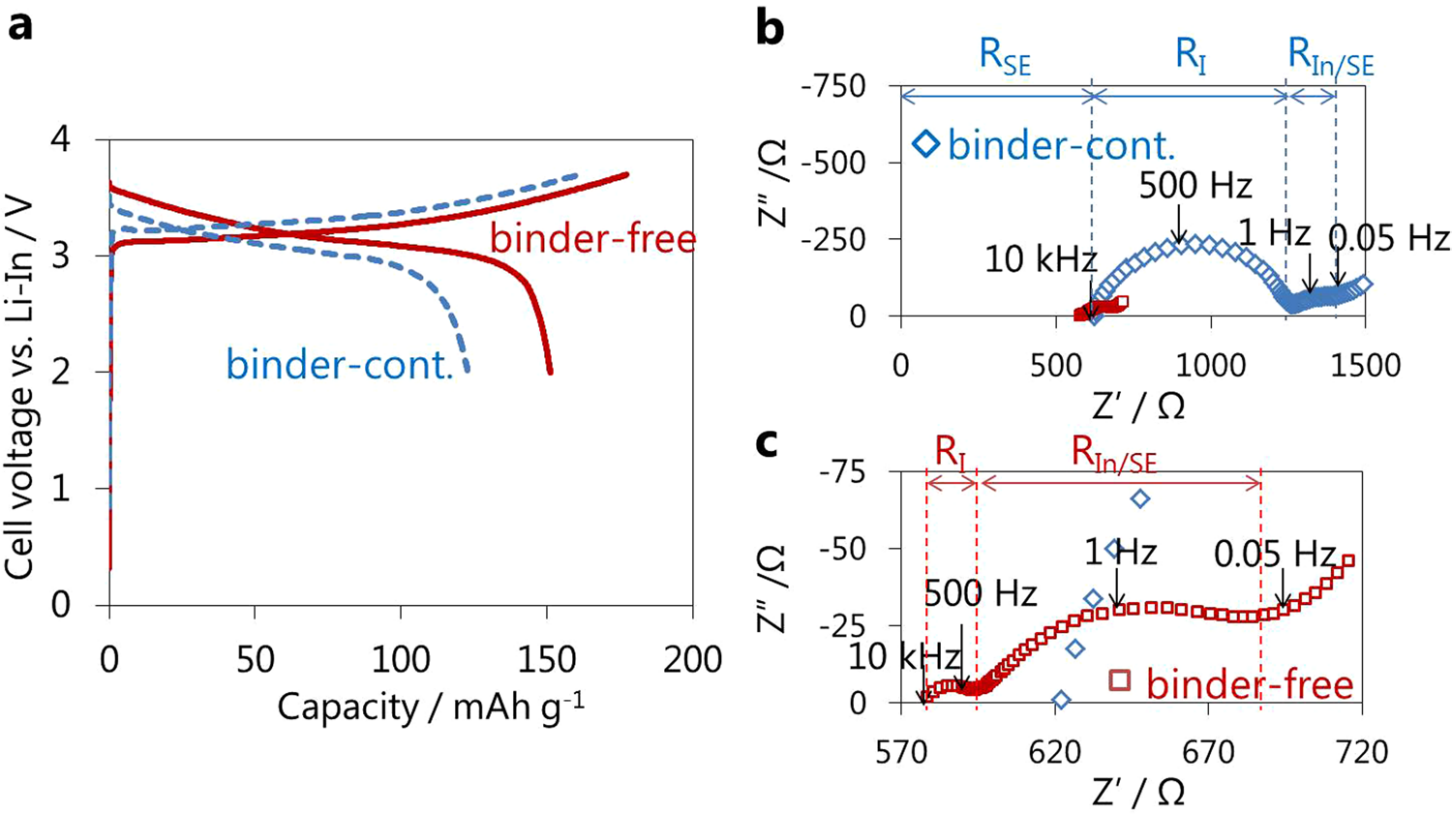
Efficacy of the removal of PPC from positive half-cells. (**a**) Initial charge-discharge curves of binder-free and binder-containing positive half-cells (NCM:f-LPS:AB:PPC = 80:20:2:3)/f-LPS/In operated at a current density of 0.064 mA cm^−2^ between the cut-off voltage of 3.7–2.0 V vs. Li-In and (**b**), (**c**) the corresponding impedance plots measured after charging to 3.7 V vs. Li-In.

Figure 3a compares the initial charge-discharge curves of binder-containing and binder-free positive half-cells constructed using as-prepared 3 wt.% PPC-containing positive sheets, which had average discharge voltages of 3.04 and 3.15 V and discharge capacities of 123 and 151 mAh g^−1^, respectively. The binder-free discharge capacity corresponded to the substantial theoretical capacity of NCM^25^. Impedance measurements were performed to clarify the differences in cell performance (Fig. 3b,c). The resistance at the intersection with the real axis around 10 kHz represents the bulk and grain boundary resistance of the SE layer (R_SE_)^26^. The resistance at the semicircles with peak-top frequencies of ~500 and 1 Hz correspond to the charge transfer resistance of NCM/SE (R_I_) and In/SE interface (R_In/SE_), respectively^26^. While PPC was not used in the SE layer, the R_SE_ value was reduced by ~40 Ω by heat treatment, implying a decrease in the grain boundary and void volume by heating to temperatures near the glass transition temperature of LPS^19,20^. The R_I_ value was drastically reduced from 638 Ω to 14 Ω by the removal of PPC, suggesting an increased contact area between NCM and the SE.

**Figure 4:**
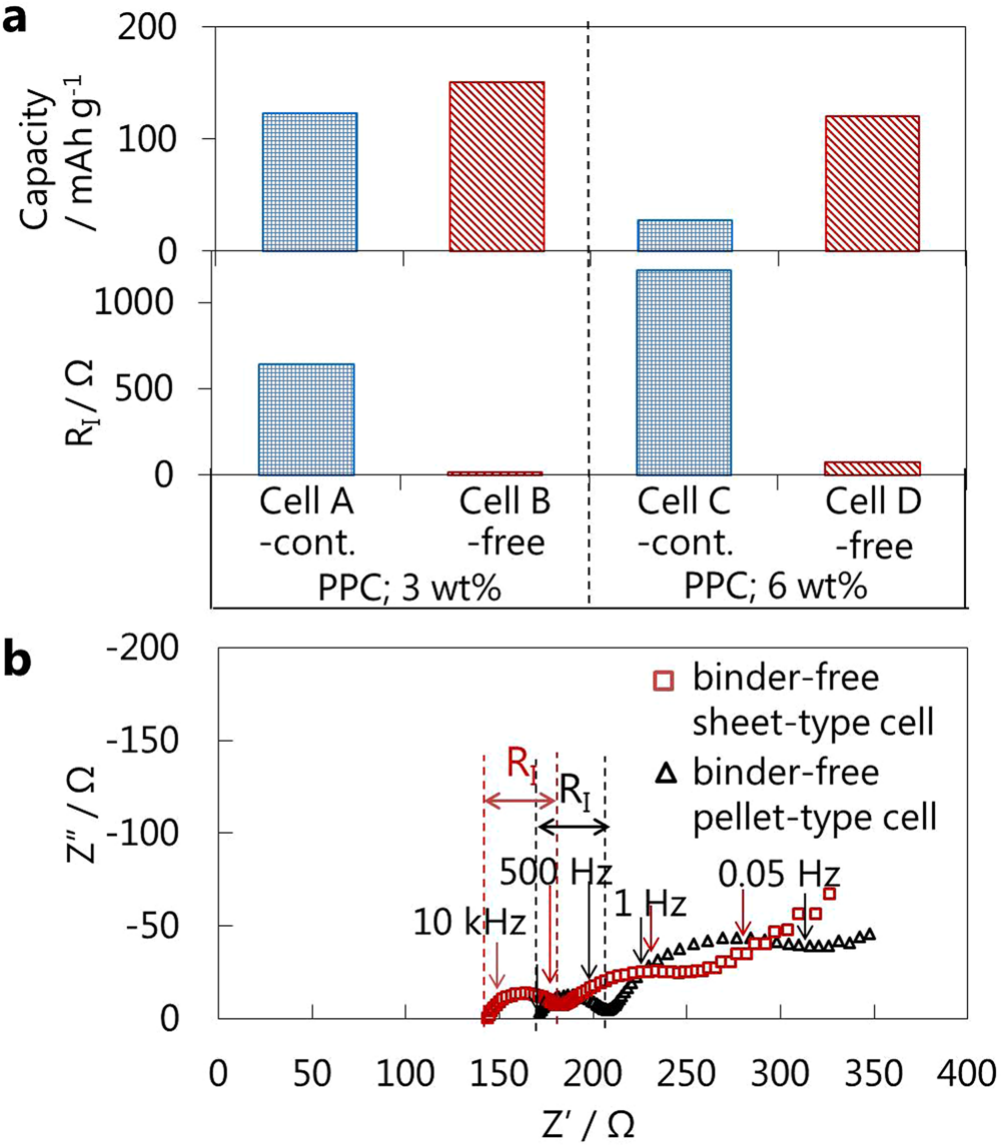
Effect of PPC content in the electrode on the cell performance. (**a**) Initial discharge capacities and R_I_ after initial charging of binder-free and binder-containing positive half-cells using positive sheets with different PPC content, (NMC:f-LPS:AB:PPC = 80:20:2:x)/LPS/In. (x = 3, 6) (**b**) Impedance plots of the binder-free sheet-type cell, sheet (NCM:f-LPS:AB:PPC = 80:20:4:3)/LPS/In, and the binder-free pellet-type cell, powder (NCM:f-LPS:AB = 80:20:4)/LPS/In, measured after initial charging to 3.7 V vs. In-Li at a current density of 0.064 mA cm^−2^.

The effect of PPC content on the positive sheets was investigated for binder-containing and binder-free positive half-cells using positive sheets containing 3 wt.% and 6 wt.% PPC (Fig. 4a). The R_I_ of binder-containing half-cell A (PPC: 3 wt.%) was half that of binder-containing half-cell C (PPC: 6 wt.%), increasing the capacity. Binder removal induced drastic decreases in R_I_, resulting in improved capacity for both cells. Compared to binder-free half-cell D (PPC: 6 wt.%), the binder-free half-cell C (PPC: 3 wt.%) exhibited a higher discharge capacity and one-fifth as much R_I_. The small R_I_ indicates a large contact area between NCM and the SE. When the volume ratio of PPC/SE in the as-prepared positive sheet (PPC/SE = 0.22 (PPC: 3 wt.%), PPC/SE = 0.45 (PPC: 6 wt.%)) is small enough, voids and cracks between NCM and the SE originating from the removal of PPC can be filled completely during the plastic deformation of the SE by compression. The R_I_ value for the binder-free sheet-type half-cell (PPC; 3 wt.%) was consistent with that of a binder-free pellet-type half-cell, suggesting a similar NCM and SE contact area was achieved by binder removal (Fig. 4b). Conversely, when the PPC content was too low (around 1 wt.%), the positive sheets crumbled easily. Although the PPC content should be adjusted by the specific surface area of the components and composition ratio of active materials, SE, and conductive additive, in this case, a PPC content of around 3 wt.% is desirable for manufacturing positive sheets.

**Figure 5:**
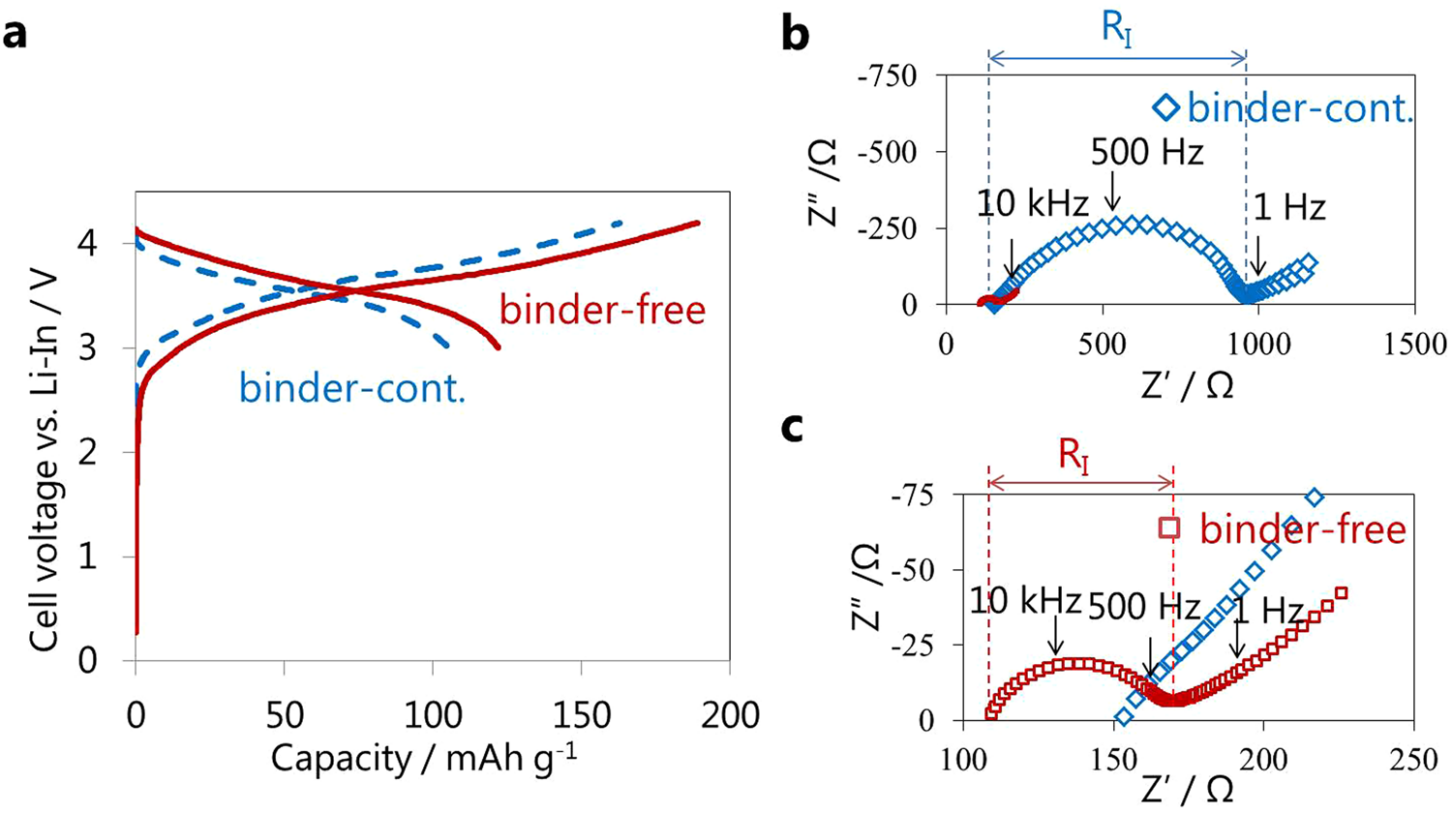
Efficacy of the removal of PPC from full-cells. (**a**) Initial charge-discharge curves of binder-free and binder-containing full-cells, (NCM:f-LPS:AB:PPC = 80:20:2:3)/(LPS:PPC = 100:3)/(graphite:f-LPS:AB:PPC = 58:42:1:3) operated at a current density of 0.064 mA cm^−2^ with a cut-off voltage of 4.2–3.0 V and (**b**,**c**) the corresponding impedance plots measured after charging to 4.2 V.

In addition to the improved positive half-cell performance, the binder removal process also improved the charge-discharge capacity of negative half-cells using as-prepared 3 wt.% PPC-containing negative sheets (see Supplementary Fig. S5). The initial charge capacity increased from 257 to 308 mAh g^−1^, which was close to the theoretical value for graphite of 330 mAh g^−1^ estimated for conventional LIBs^27^. The binder removal process was further applied to sheet-type full-cells constructed using an as-prepared SE sheet containing 3 wt.% PPC (~180 μm in thickness) and positive/negative sheets (Fig. 5). Binder removal remarkably improved the average discharge plateau from 3.54 V to 3.62 V, and the discharge capacity from 103 mAh g^−1^ to 122 mAh g^−1^ (Fig. 5a). The corresponding impedance profiles show a decrease in R_SE_ (Fig. 5b,c), suggesting that the removal of PPC from the SE layer induces a decrease in the grain boundary and void volume^19,20^. As for the positive half-cells (Fig. 3b,c), R_I_ for the full-cell was also substantially reduced by the removal of PPC from 836 Ω to 60 Ω.

### Improved rate capability and stable cycling for binder-free cells

**Figure 6:**
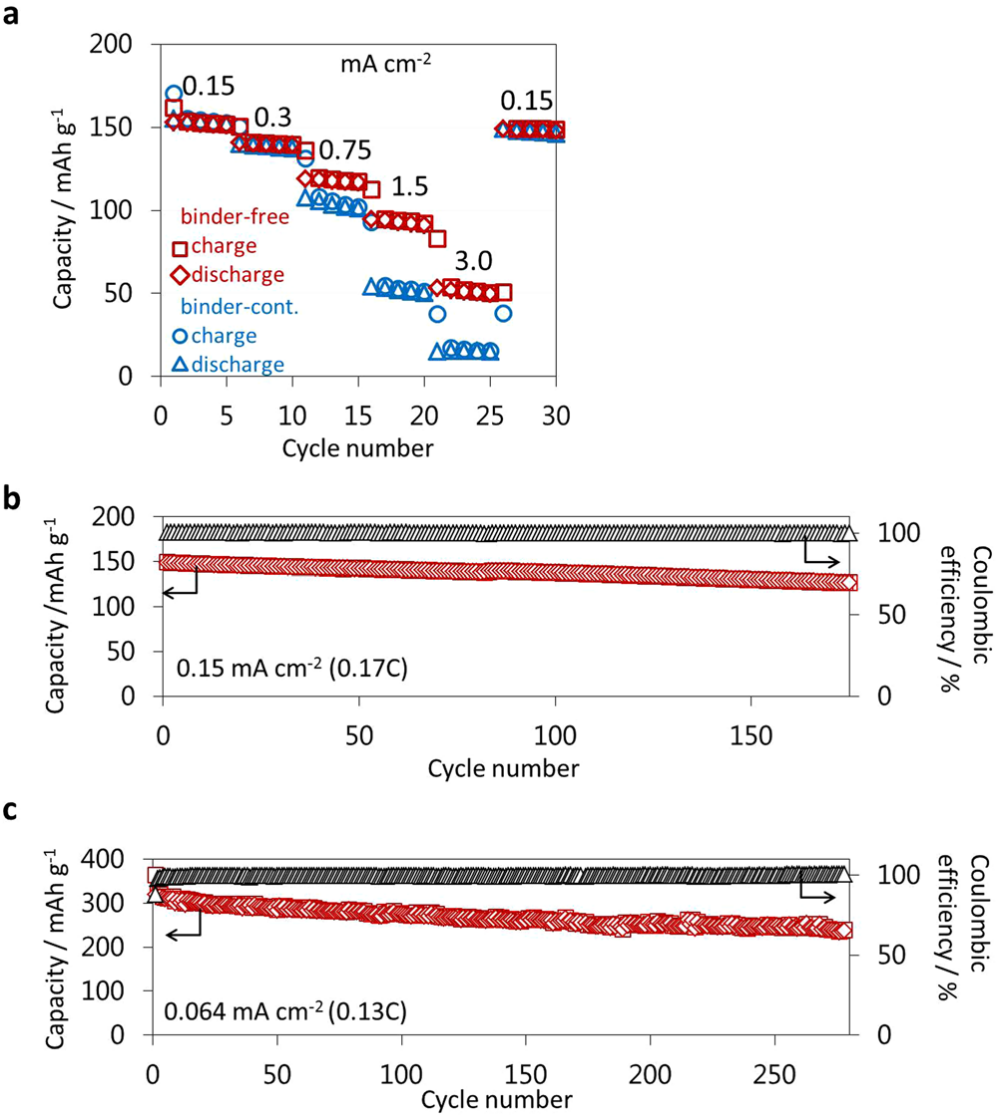
Improved rate and cycle performances of binder-free half-cells. (**a**) Rate capability of the binder-free and binder-containing positive half-cells, (NCM:f-LPS:AB:PPC = 80:20:4:3)/LPS/In-Li, operated at several current densities from 0.15 mA cm^−2^ (0.17 C) to 3.0 mA cm^−2^ (3.5 C) under CCCV charge-CC discharge between 3.7 and 2.0 V (vs. Li-In) at 30 °C. The numbers denote the operating current densities in mA cm^−2^. Cycling retention of (**b**) binder-free positive half-cell shown in (**a**) operated at current density of 0.15 mA cm^−2^ (0.17 C) under CCCV charge-CC discharge and (**c**) binder-free negative half-cell, (graphite:LS-LPS:AB:PPC = 58:42:2:3)/LPS/In-Li, operated at 0.064 mA cm^−2^ (0.13 C) under CC charge-CC discharge.

Figure 6a compares the rate capability of binder-free and binder-containing positive half-cells. Under a low current density of 0.15 mA cm^−2^, both cells show slight differences in their measured capacities (153 mAh g^−1^ for the former, 155 mAh g^−1^ for the latter), comparable with the theoretical value of NCM^25^. The use of milder measurement conditions, such as CCCV-charge mode and an In-Li counter electrode, caused the increased initial capacity in the latter cell, compared to Fig. 3a. Notably, with increasing current densities, the binder-containing cells display rapid decays of capacities and negligible capacities, 15 mAh g^−1^, starting at 3.0 mA cm^−2^. In contrast, the binder-free cell exhibited significantly higher capacity, delivering ~50 mAh g^−1^ even at 3.0 mA cm^−2^. Further cycling at a low current density returned the cell to a reversible capacity of 149 mAh g^−1^. Thus, we concluded that the rate capability is remarkably improved by reducing the R_I_ value.

Stable charge/discharge cycling was exhibited by binder-free positive half-cells after 25 cycles at the above testing rate (Fig. 6b). The cell shows an initial discharge capacity of 149 mAh g^−1^ with 100% coulombic efficiency and retains a high capacity of greater than 125 mAh g^−1^ for at least 175 cycles. The capacity retention rate of binder-free and binder-containing half-cells that were operated at several current densities are shown in Supplementary Fig. S6a, which obtained from the discharge capacity of N^th^ cycle compared to that of (N-4)^th^ cycle in Fig. 6a. The binder-free cell operated at 0.15 mA cm^−2^ had a capacity retention rate of 99.4% per 5 cycles (30^th^/26^th^). Thus, the capacity after the 175^th^ cycle was estimated to be 121 mAh g^−1^ (see Supplementary Fig. S6b), which is comparable with the experimental value (125 mAh g^−1^) in Fig. 6b. This agreement suggests the capacity retention rate per 5 cycles may be a reliable measure for estimating the long-term stability under various current densities. The capacity retention rates of the binder-free cell decreased with increasing current densities (see Supplementary Fig. S6a). This decrease can be improved by reducing the dominant cell internal resistance for the half-cell, R_SE_, by using a thinner SE layer, a SE with a higher ionic conductivity (~10^−2^ S cm^−1^)^10^, and higher-temperature operation (~100 °C)^10^. In comparison with a binder-containing cell, the binder-free cell maintains higher capacity retention rates at every current density (see Supplementary Fig. S6a). Therefore, the reduction of R_I_ by the removal of the binder is effective to enhance long-term stability especially for the operation under higher current densities. Stable cycling was also exhibited for binder-free negative half-cells (Fig. 6c). The cell shows an initial charge capacity of 319 mAh g^−1^ with 88% coulombic efficiency. Notably, extended testing for more than 250 cycles indicated excellent reversible capacity above 240 mAh g^−1^ with coulombic efficiencies in excess of 99% after a few initial cycles.

### Fabrication of binder-free sheet-type full-cells with high cell-based-energy-density

We fabricated SE sheets with half their original thickness and positive/negative sheets with twice the original loading of active materials to improve the cell-based energy density. Moreover, the capacity ratio of negative to positive sheets, N/P, was tuned to be ~1.2–1.5 to prevent the occurrence of fine short-circuiting associated with the formation of lithium dendrites on the negative layer, although N/P is generally tuned to 1.1 in the industrial production of LIBs. As a result, the cell-based-energy-density of sheet-type binder-free full-cells reached 115 Wh kg^−1^, which was calculated from the initial discharge capacity (116 mAh g^−1^), the average discharge voltage (3.51 V), and the weight of the cell without current collectors (PE 16.38 mg, SE 10.93 mg, NE 16.89 mg) (see Supplementary Fig. S7).

**Figure 7:**
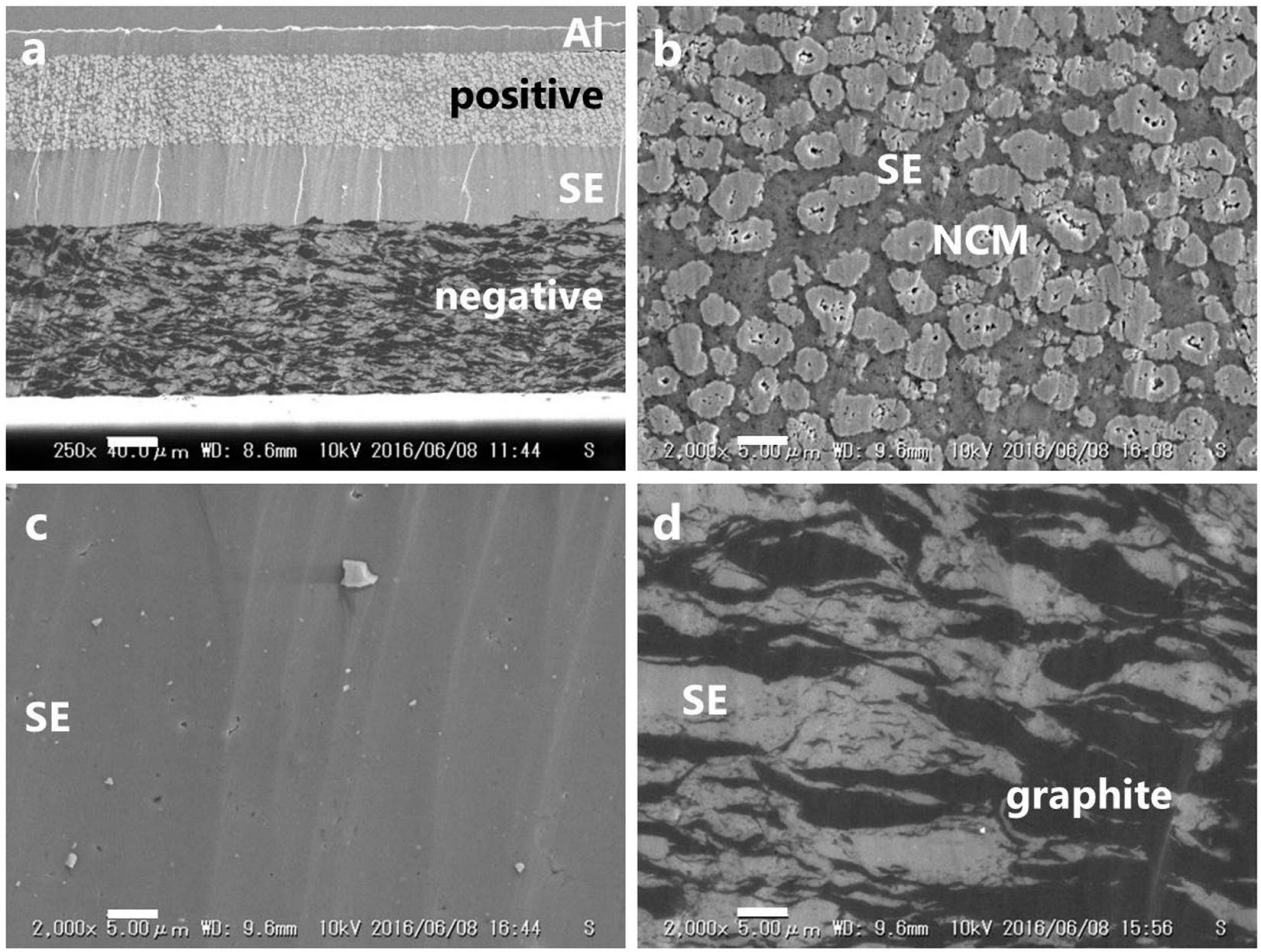
SEM images of intimate contact of SE and positive/negative layer maintained over 350 cycles. Cross-sectional SEM images of (**a**) the binder-free full-cell, (NCM:LS-LPS:AB:PPC = 80:20:2:3)/(LPS:PPC = 100:3)/(graphite:LS-LPS:AB:PPC = 58:42:2:3), and the magnified images of the (**b**) positive layer, (**c**) SE layer and (**d**) negative layer after 350 cycles. Scale bar, (**a**) 40 μm, (**b**–**d**) 5 μm.

The above binder-free sheet-type full-cell after 350 cycles could be taken out from the mold without breaking, indicating its free-standing feature, and its microstructure was observed by SEM (Fig. 7). The thickness of the positive layer, the SE layer, and the negative layer were estimated to be 74, 59, and 134 μm, respectively (Fig. 7a). Despite a relatively thin SE layer, the positive and negative sheets were successfully stacked on both sides of the SE sheet without short-circuiting. NCM particles were dispersed in the SE matrix homogeneously and made intimate contact with the SE (Fig. 7b). In addition, no pulverization of NCM by compression between NCM particles^14^ was observed in the cross-sectional view of the positive layer. The SE layer appears as an almost completely dense region without voids, cracks, or grain boundaries (Fig. 7c). Similarly, smooth cross-sections were also observed in hot-pressed SE pellets^19,20^. Even without added pressure, sintering of SE particles may be promoted by heat treatment near the glass transition temperature. The originally spherical graphite powder was crushed with cleavage in the direction perpendicular to the external pressure used in the fabrication of the cell to form a closely packed composite with the SE particles (Fig. 7d). Scarcely any evidence of PPC removal, such as visible voids and cracks in the electrode and electrolyte layers, was found by SEM. Surprisingly, the active materials and SE make intimate contact and are densely packed even after 350 cycles and despite graphite’s ~10 vol% volume change^28^.

## Discussion

Our work demonstrates the novel fabrication process of sheet-type ASSLBs in which the binder can be removed. Most of the conventional binders for LIBs, such as PVdF, and for sheet-type sulfide-based ASSLBs, such as silicone^12^, SEBS^14,15^, SBS^15^, PEG^16^, and BR^17^ decompose around 400 °C under an oxygen-free atmosphere causing carbon deposits, which may affect the electronic conductivity of the separator layer. Therefore, we focused on volatile aliphatic polycarbonates, which depolymerize by unzipping at lower temperatures. The aliphatic polycarbonates tend to be hard to dissolve in solvents with low donor number, while SE tends to be decomposed by solvents with a high donor number. A combination of PPC and anisole fulfilled these conflicting demands.

PPC acts as a suitable dispersant and binder to make homogeneously dispersed slurries that afford bendable free-standing SE sheets, splinter-free electrodes during punching, and two-layered SE/electrode sheets by coating. The removal of PPC even from large-area SE sheets with high PPC content induces no adverse effects on the conductivity of the SE, implying the possibility of preparing large-area sheet-type cells which are important for applications. The binder-free sheet-type full-cells were also free-standing, similar to the previously reported binder^12,15^, polymer mesh^12^ and nonwoven^13^-containing sheet-type cells. This property was accomplished by the intimate contact between the SE and the active materials through the plastic deformation of SE^19,20^ by compression after heat-treatment. Therefore, the SE can act as a binder after the removal of PPC.

The binder removal strategy is a simple, effective, and fruitful approach to producing sheet-type cells. To the best of our knowledge, this report is the first case that realizes binder-free sheet-type sulfide-based ASSLBs by coating.

Long-term stability of the binder-free half-cells was achieved despite operation under much harsher conditions relative to previously reported pellet-type half-cells^7–9^, including twice the ratio of positive active materials in the electrode and a two-fold increase in current density. Although previously reported binder-containing half-cells were investigated for only 20 cycles or less^12,14,15^, and some battery exhibits significant capacity fading^12^, we validated the reliability of the binder-free half-cells over 175 cycles. While gradual capacity fading for the binder-free full-cell was observed over 350 cycles, this may be improved by using a SE with high ionic conductivity and high operating temperatures^10^. Work to enhance the reversible cycle stability of these full-cells is currently in progress.

Binder removal drastically decreases R_I_, which is lower than that reported for SEBS^14,15^ and SBS-containing cells^15^, and reaches values close to those seen for binder-free pellet-type cells (Fig. 4c). As a result, the rate capability (Fig. 6a) and capacity retention rates (see Supplementary Fig. S6a) were improved by binder removal, especially under higher current densities. As the thickness of the SE layer decreases and the conductivity of SE (10^−2^ S cm^−1^)^10^ increases, R_I_ is expected to become the dominant factor in the total cell internal resistance, comprising R_I_ and R_SE_^29^. Therefore, minimizing R_I_ is critical to improving the rate capability and long-term stability, and is effectively accomplished by removing the binder from sheet-type cells.

Stackable binder-free sheet-type batteries may potentially satisfy the demands of next-generation electronic vehicles in terms of energy density. These batteries exhibited an energy density of 115 Wh kg^−1^, which is a ~2.6–5-fold increase compared with conventional pellet-type cells (LiCoO_2_/graphite)^10,11^ and a 2.6-fold increase relative to nonwoven-containing sheet-type cells (LiCoO_2_/Li_4_Ti_5_O_12_)^13^. This feat was accomplished through the fabrication of thinner SE sheets and homogeneously dispersed electrodes with higher active material weight fractions by using PPC.

The results of this study provide a novel and unique process that can enhance rate capability, long-term stability and cell-energy-density, and offer opportunities to build high-performance sheet-type ASSLBs that are not limited to NCM/graphite ASSLBs.

## Methods

### Evaluation of solvent-exposed LPS and PPC-containing LPS before and after heat treatment

All processes were performed in a dry Ar atmosphere. Three types of 75Li_2_S · 25P_2_S_5_ (Li_3_PS_4_) were prepared according to previous reports: Li_3_PS_4_ glass (LPS) prepared by mechanical milling (~10 μm in diameter, 4 × 10^−4^ S cm^−1^)^5^; pulverized fine Li_3_PS_4_ glass (f-LPS) (~3 μm in diameter, 2 × 10^−4^ S cm^−1^)^30^; thin rod-like Li_3_PS_4_ (LS-LPS) prepared by liquid-phase shaking (LS) (ca. 3 μm × 500 nm × 100-200 nm, 2 × 10^−4^ S cm^−1^)^31,32^. The stability of LPS toward solvents was evaluated by soaking LPS powder in a solvent for 24 h followed by drying for a few days at room temperature. PPC-containing LPS was prepared by mixing LPS and PPC (M_w_ = 357,000, PO units = 3 wt.%, Sumitomo Seika Chemicals) in a weight ratio of 100:6 in anisole and drying the resultant slurry at room temperature for a few days in a dry Ar atmosphere. Heat treatment of the resultant powder was carried out in a stainless-steel container on a hot plate under vacuum for 30 min.

The ionic conductivities of pelletized samples were measured by AC impedance spectroscopy at frequencies ranging from 0.1 Hz to 1 MHz under an applied voltage of 10 mV (FRA1455, Solartron) in a symmetric cell (stainless-steel/sample/stainless-steel). The structure was studied by Raman spectroscopy with a green laser at 532.05 nm (NRS-3100, JASCO). Cyclic voltammetry measurements were conducted to quantify the voltage stability window for the pelletized sample using Li foil as the counter/reference electrode, a stainless-steel disk as the working electrode, and a sweep rate of 5 mV s^−1^ between −0.1 to 5 V. DC conductivity was measured using a potentiostat (Multistat 1470E, Solartron) at room temperature under a constant DC voltage of 50 mV in a symmetric cell configured as either Li/sample/Li or stainless-steel/sample/stainless-steel, and calculated from the thickness of the pellets and obtained current values.

### Fabrication of binder-free sheet-type ASSLBs

Typical electrode and SE sheets were prepared by mixing the active materials LiNbO_3_-coated LiNi_1/3_Co_1/3_Mn_1/3_O_2_ (NCM)^33^ and graphite (Nippon Graphite, CGB-10), f-LPS, acetylene black (AB, Denki Kagaku, Denka Black HS-100), and PPC in anisole with a weight ratio of 80:20:2:3 (NCM:f-LPS:AB:PPC), 58:42:1:3 (graphite:f-LPS:AB:PPC) and 100:3 (SE:PPC), casting the resultant slurry onto aluminum foil (20 μm) or copper foil (18 μm), and drying at room temperature (see Supplementary Fig. S8). Two-layered SE/electrode sheets were also prepared by coating an SE slurry on the electrode sheet.

Typical half-cells were fabricated using the electrode sheet as the working electrode, SE glass powder as the separator, and In foil or In-Li foil as the counter and reference electrodes. The electrode sheet and SE powder (80 mg) were placed in a 10 mm-diameter polyimide mold and pressed together by applying a pressure of 110 MPa^14^. Then, the bilayer sheet was placed in a stainless-steel container and heated at 225 °C for 30 min under vacuum. An In foil or In-Li foil were placed on the surface of the SE side of the bilayer sheet and compressed using two stainless-steel rods as current collectors under ~100 MPa. The sheet-type full-cells were constructed as described above except for simultaneous stacking of the PPC-containing positive, the SE, and the negative sheet.

### Evaluation of the binder-free sheet-type ASSLBs

Charge-discharge cycling was conducted using a charge-discharge measurement device (BTS-2004, Nagano) between cut-off voltages of 2.0–3.7 V vs. Li-In for the positive half-cells, −0.57–0.88 V vs. Li-In for the negative half-cells, and 3.0–4.2 V for the full-cells at room temperature. The capacity calculations are based on the mass of NCM for positive half-cells and full-cells, and graphite for negative half-cells. AC impedance measurements were performed after initial charging at a current density of 0.064 mA cm^−2^ under frequencies ranging from 0.01 Hz to 1 MHz at an applied voltage of 10 mV.

The morphology and microstructure of the full-cell were characterized by scanning electron microscopy (KEYENCE VE-9800) at 10 kV. Cross sections of the specimens were formed by Ar milling using a cooling cross section polisher (CP, JEOL 1B-19520CCP). The samples were transferred in an Ar atmosphere from the glove box to the equipment for CP and SEM.

## Electronic supplementary material

Supplementary
