# Supplementary material for: Binder-free sheet-type all-solid-state batteries with enhanced rate capabilities and high energy densities
Source: Sci Rep. 2018 Jan 19;8:1212. doi: 10.1038/s41598-018-19398-8 (PMC5775432; doi:10.1038/s41598-018-19398-8)
Supplement: Supplementary file 1 — Supplementary [file 41598_2018_19398_MOESM1_ESM.pdf]

Supplementary

Binder-free sheet-type all-solid-state batteries with enhanced rate capabilities and high energy densities

Mari Yamamoto[*a], Yoshihiro Terauchi[a], Atsushi Sakuda[b], Masanari Takahashi[ac]

[a] Osaka Research Institute of Industrial Science and Technology, Morinomiya Center, 1-6-50, Morinomiya, Joto-ku, Osaka-city, Osaka 536-8553, Japan

[b] Department of Applied Chemistry, Osaka Prefecture University, 1-1 Gakuen-cho, Naka-ku, Sakai, Osaka 599-8531, Japan

[c] Graduate School of Materials Science, Nara Institute of Science and Technology, 8916-5 Takayama-cho, Ikoma, Nara 630-0192, Japan

* Tel.: +81-6-6963-8085. Fax: +81-6-6963-8099. E-mail: mari@omtri.or.jp

Supplementary Fig. S1

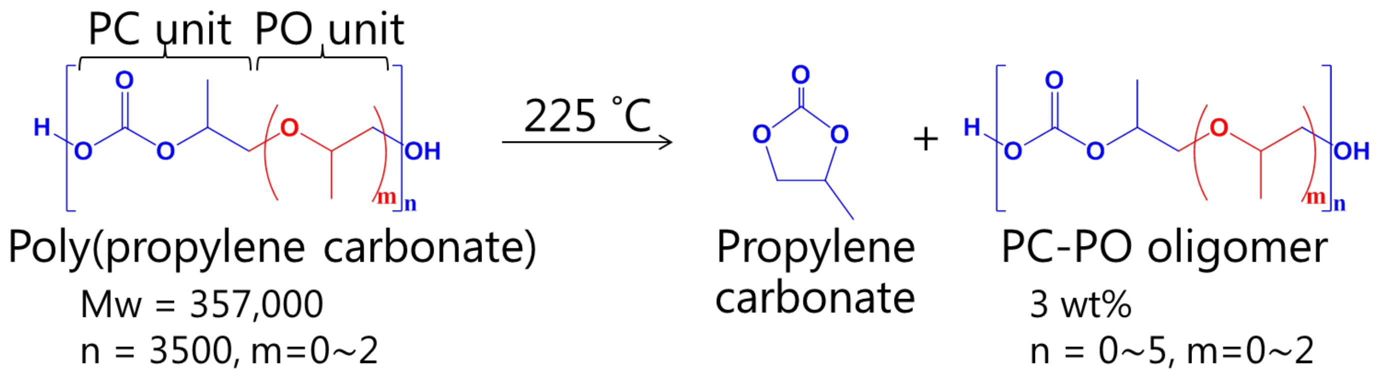

Supplementary Fig. S1| Depolymerization of poly(propylene carbonate) by unzipping at 225 ˚C to afford propylene carbonate which quickly evaporate under vacuum. Consecutive propylene oxide units (ether linkages) in the backbone are stable at 225 ˚C to afford propylene carbonate-propylene oxide (PC-PO) oligomer.

Supplementary Table S1

Aliphatic polycarbonates	Poly(ethylene carbonate) (PEC)	Poly(propylene carbonate) (PPC)
	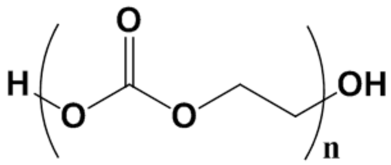	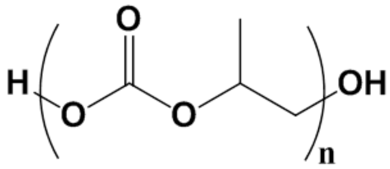
M_w ($\times 10^3$)	190	357
Decomp. temp. (˚C)	225	225
Decane	insoluble	insoluble
1,2-Dichloroethane	soluble	soluble
Toluene	insoluble	insoluble
Anisole	insoluble	soluble

Supplementary Table S1| Solubility of aliphatic polycarbonates in the solvents.

Supplementary Table S2

Positive electrode sheet<br><br>NCM:f-LPS:AB:binder<br>=80:20:1:3 (in wt. ratio)	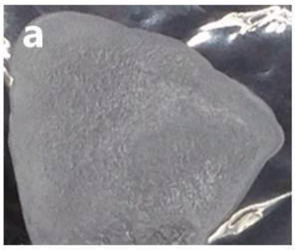	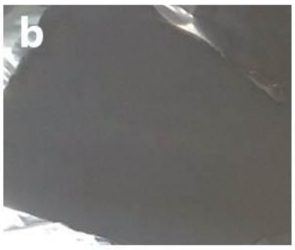
Binder	PEC	PPC
Solvent	1,2-Dichloroethane	Anisole
Vapor pressure / kPa [23]	8.7 (20 ˚C)	0.47 (25 ˚C)

Supplementary Table S2| Positive sheets fabricated using (**a**) PEC/1,2-dichloroethane and (**b**) PPC/anisole with NCM, f-LPS, AB and binder in the weight ratio of 80:20:1:3. The sheet in (**a**) has a whitish surface indicating the floating of light SE powder and bulges due to the rapid evaporation of the solvent, while the sheet shown in (**b**) has a homogeneous dark surface as anisole has a sufficiently low vapor pressure.

Supplementary Fig. S2

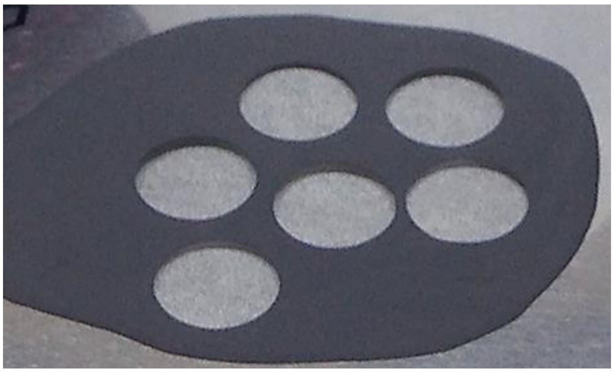

Supplementary Fig. S2| Photo of a positive electrode sheet (NCM:f-LPS:AB:PPC = 80:20:1:3) punched via 5 months after fabrication.

Supplementary Fig. S3

Heat treatment temperature /˚C	25	225	250	290
LPS	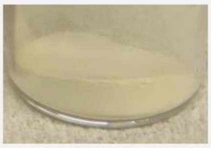	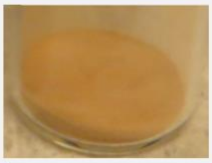	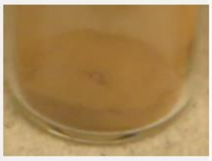	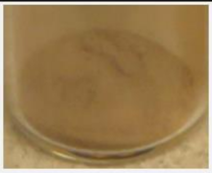
PPC-cont. LPS	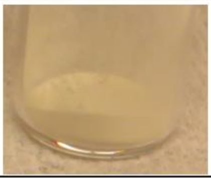	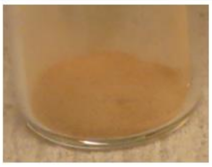	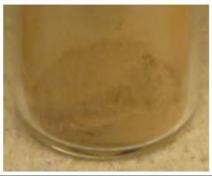	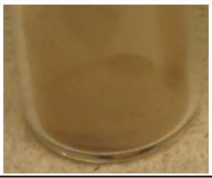

Supplementary Fig. S3| Photos of LPS and PPC-containing LPS treated at various temperatures. Similar color changes from pale yellow to dark brown are observed for both samples as the temperature is raised, indicating that the removal of PPC has no effect on the color change.

Supplementary Fig. S4a

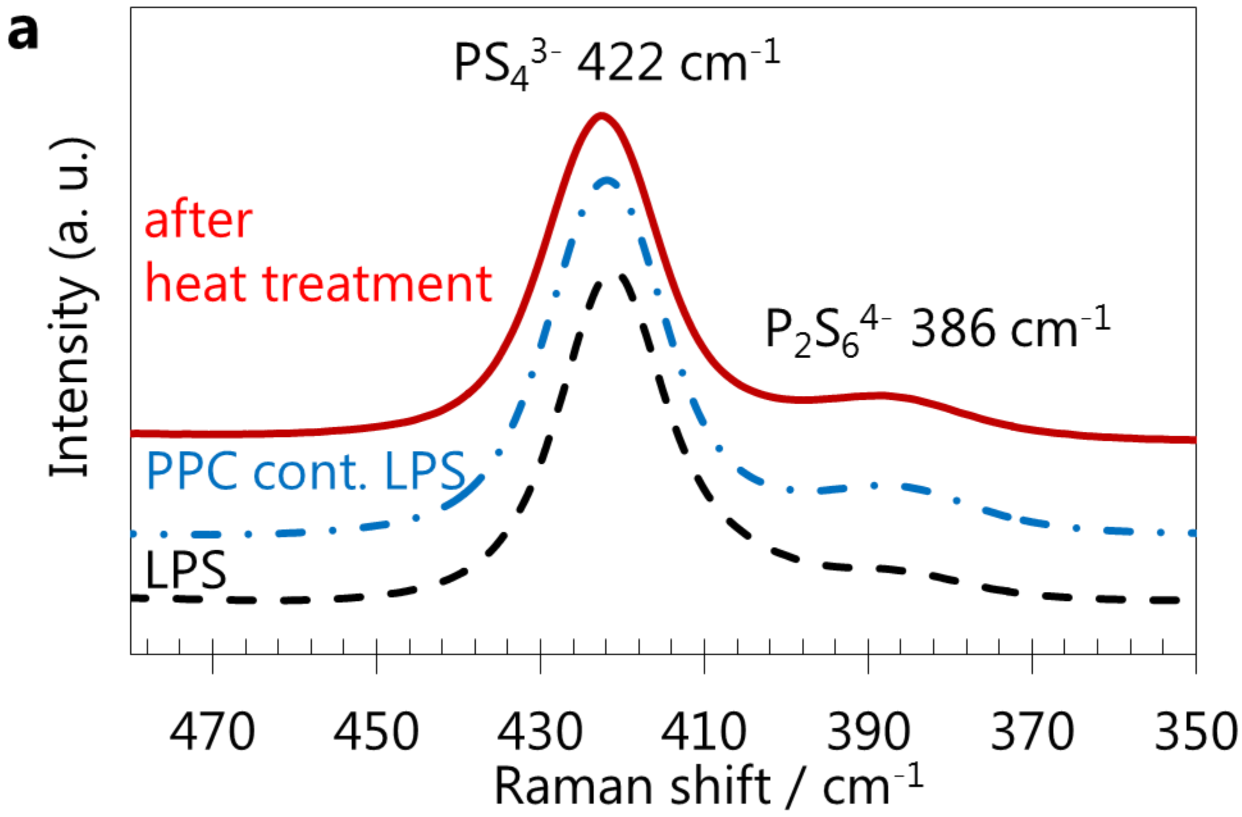

Supplementary Fig. S4 | **Influence of heat treatment on PPC-containing LPS**

(**a**) Raman spectra of original LPS and PPC-containing LPS before and after heat treatment at 225 °C. PPC-containing LPS exhibit main bands at 422 cm^{-1} and a weak band at 386 cm^{-1} before and after heat treatment, corresponding to the stretching vibration of the P-S bonds in PS_4^{3-} and $P_2S_6^{4-}$, respectively. These bands are similar to that of the original LPS glass,[21] suggesting the retention of the framework structure containing PS_4^{3-} moieties.

Supplementary Fig. S4b

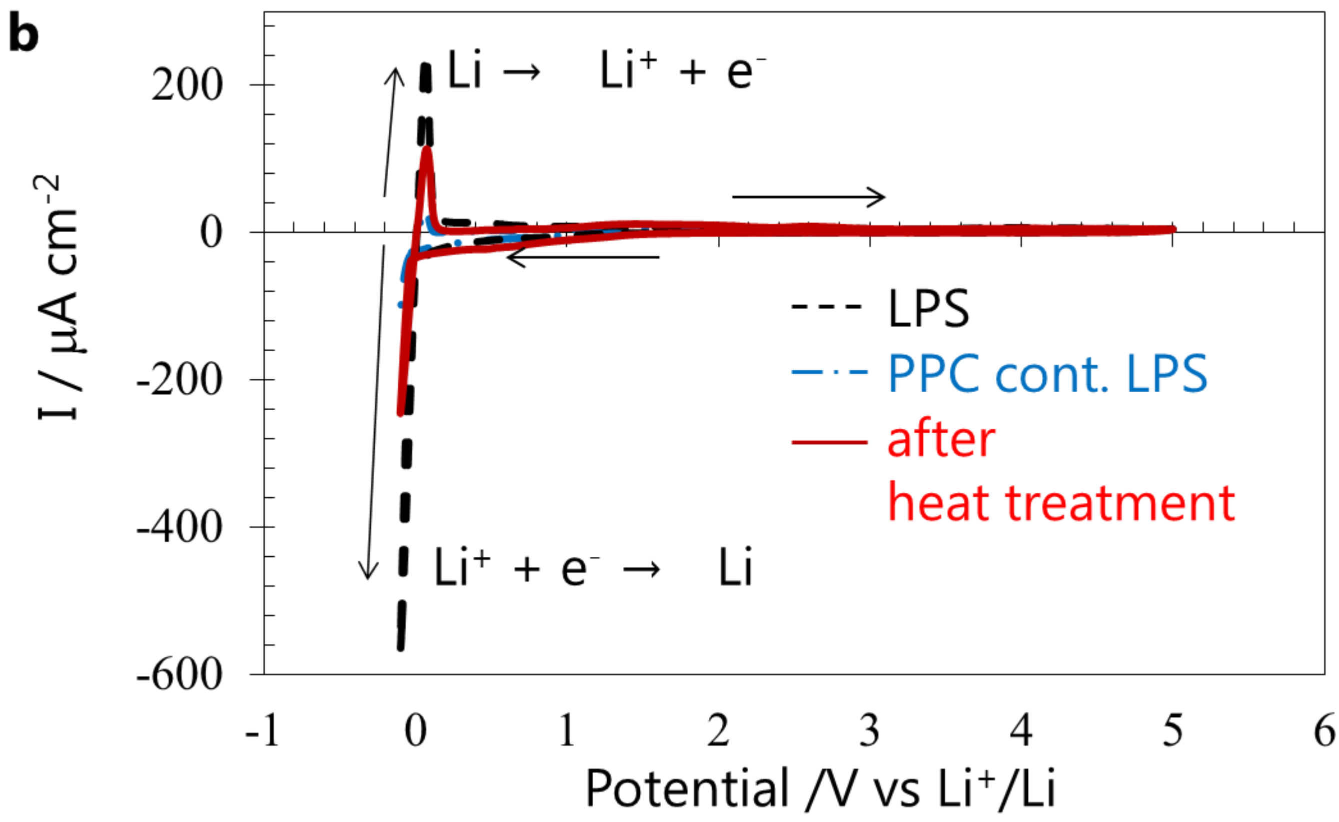

(**b**) Cyclic voltammetry (CV) curves of pristine LPS and PPC-containing LPS before and after heat treatment at 225 °C. A stainless-steel disk as the working electrode and a lithium foil as the counter/reference electrode was used. The potential sweep was performed with a scanning rate of 5 mV s^{-1} in the range from -0.1 to 5 V at room temperature. Similar to the original LPS, reversible Li deposition and dissolution currents were observed at about 0 V versus Li^+/Li, and no remarkable oxidation and reduction currents were observed in the range from 0.2 V to 5 V, suggesting a wide electrochemical window of 5 V and electrochemical stability against Li metal.

Supplementary Fig. S4c

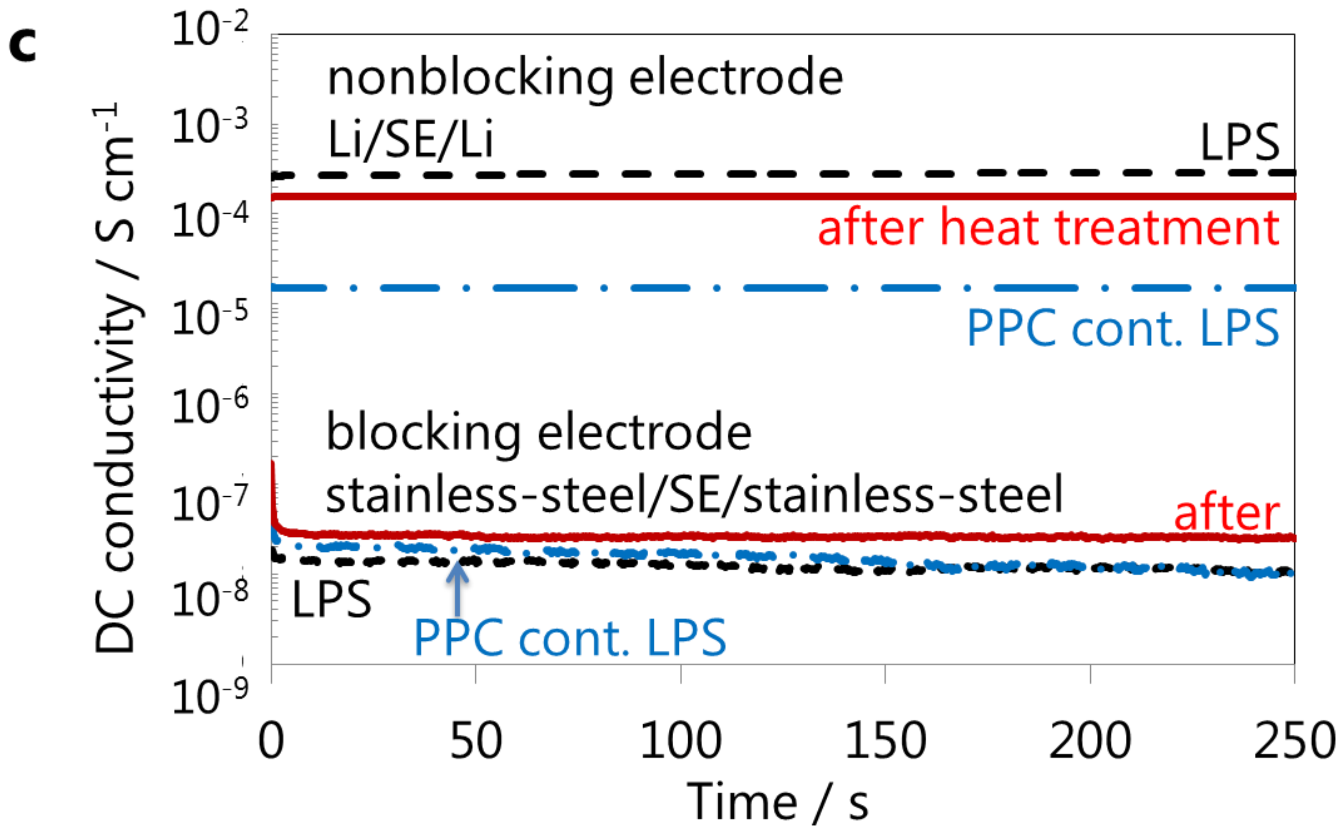

(**c**) Time dependence of DC conductivities calculated from the current obtained by applying a constant DC voltage of 50 mV to original LPS and PPC-containing LPS before and after heat treatment at 225 °C. DC conductivities obtained using lithium as non-blocking electrodes give total conductivities containing both ionic and electronic conductivity contributions, while DC conductivities obtained with stainless-steel blocking electrodes show the contribution of electronic conductivity only.

Supplementary Fig. S5

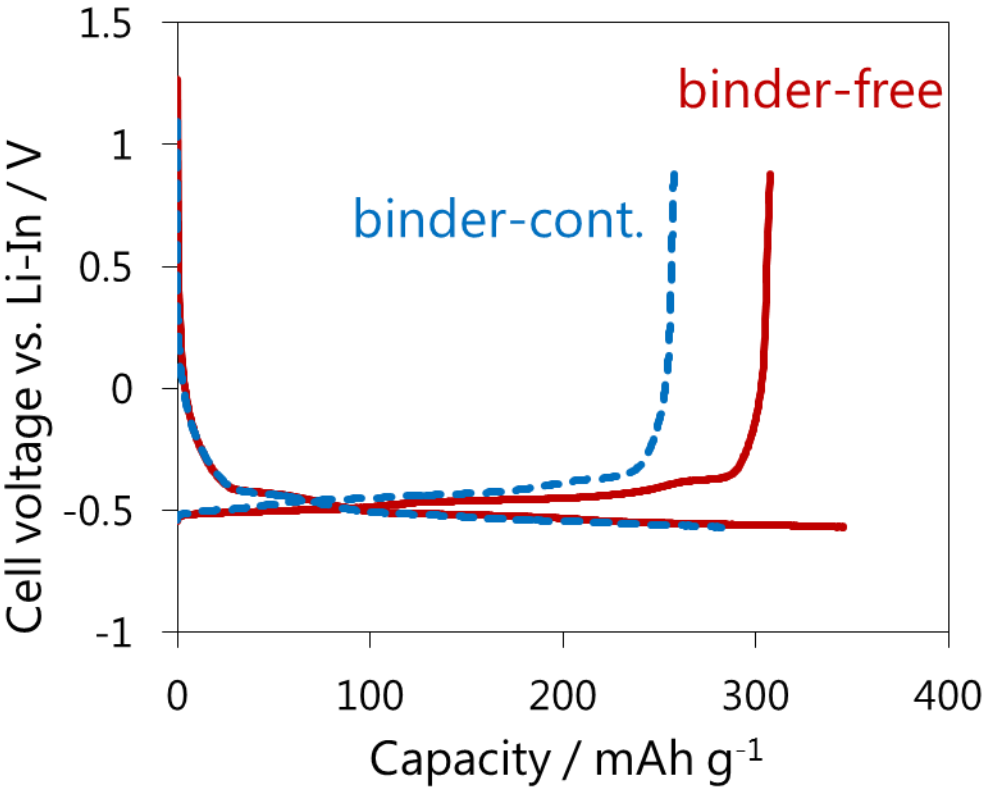

Supplementary Fig. S5| Initial charge-discharge curves of binder-free and binder-containing negative half-cells (graphite:f-LPS:AB:PPC = 58:42:1:3)/LPS/In-Li at a current density of 0.064 mA cm^{-2} and a cut-off voltage of −0.57–0.88 V vs. In-Li.

Supplementary Fig. S6

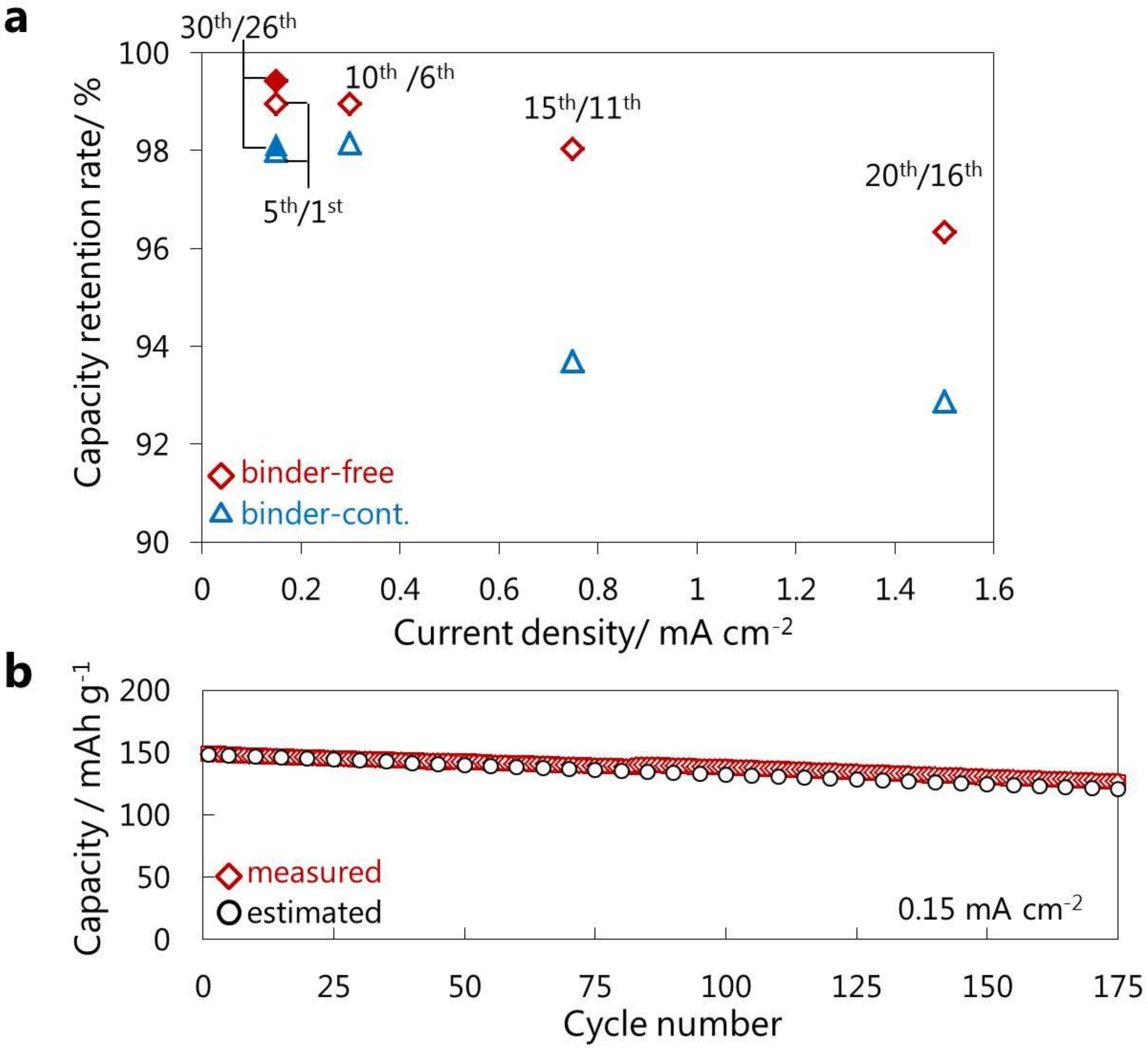

Supplementary Fig. S6| (a) Discharge capacity retention rate of Nth cycle compared to the (N-4)th cycle (Nth/(N-4)th) in the binder-free and binder-containing positive half-cells shown in Fig. 6a operated at several current densities. (b) Comparison between the estimated and measured cycle performances of binder-free positive half-cells operated at 0.15 mA cm^{-2}. Estimated values were calculated from the discharge capacity retention rate per 5 cycles at 30th/26th (0.15 mA cm^{-2}) shown in (a).

Supplementary Fig. S7

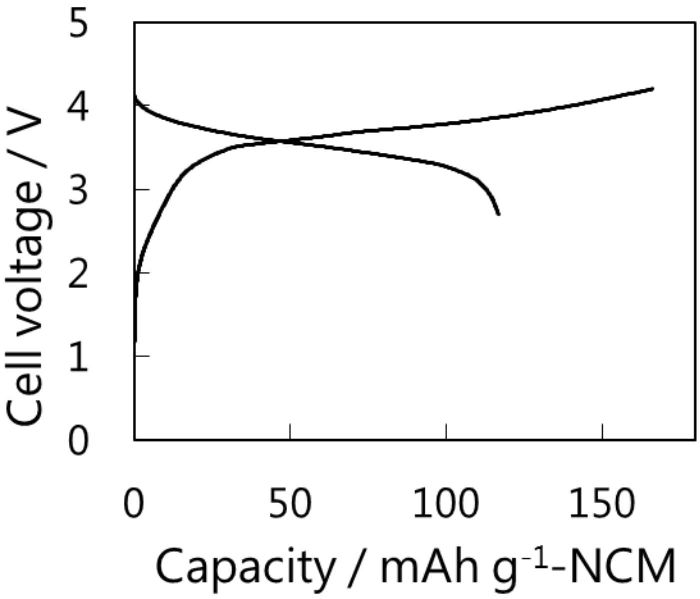

Supplementary Fig. S7| Initial charge-discharge curves of the binder-free full-cell shown in Fig. 7 constructed from a positive sheet (NCM:LS-LPS:AB:PPC = 80:20:2:3), SE sheet (LPS:SE =100:3), and negative sheet (graphite:LS-LPS:AB:PPC = 58:42:1:3 in weight ratio) at a current density of 0.064 mA cm^{-2} with a cut-off voltage of 4.2–2.7 V.

Supplementary Fig. S8

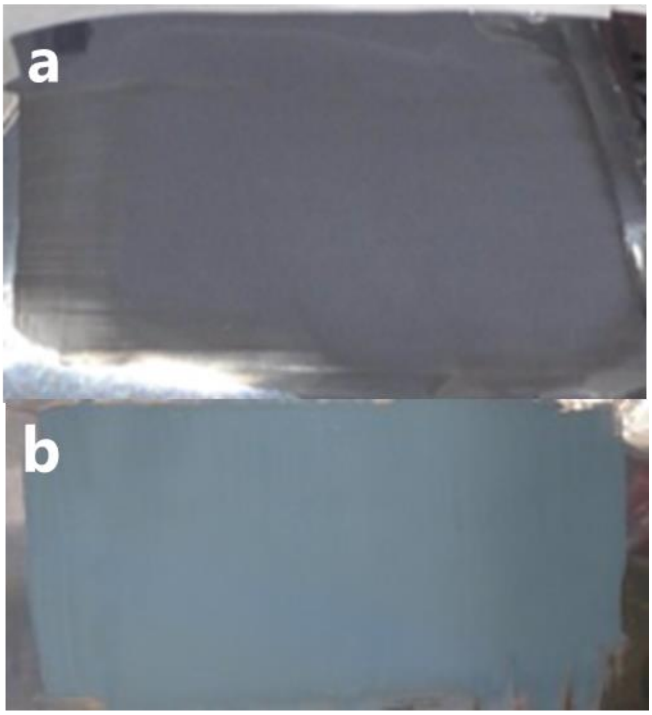

Supplementary Fig. S8| Photos of electrode sheets prepared by coating with a slurry containing (**a**) NCM:f-LPS:AB:PPC (80:20:2:3) and (**b**) graphite:f-LPS:AB:PPC (58:42:1:3) in anisole.
